# A New Therapy for Uncomplicated Vulvovaginal Candidiasis and Its Impact on Vaginal Flora

**DOI:** 10.3390/healthcare9111555

**Published:** 2021-11-16

**Authors:** Guglielmo Stabile, Roberta Marie Gentile, Stefania Carlucci, Stefano Restaino, Francesco De Seta

**Affiliations:** 1 Institute for Maternal and Child Health IRCCS “Burlo Garofolo”, 34137 Trieste, Italy; fradeseta@gmail.com; 2 Department of Medicine, Surgery and Health Sciences, University of Trieste, 34127 Trieste, Italy; Robertamarie.gentile@burlo.trieste.it; 3 Department of Obstetrics and Gynecology, Azienda Sanitaria Universitaria Giuliano-Isontina, San Polo Hospital, Gorizia-Monfalcone, 34149 Trieste, Italy; s.carlucci86@gmail.com; 4 Department of Maternal and Child Health, University-Hospital of Udine, P.le S. Maria della Misericordia n° 15, 33100 Udine, Italy; restaino.stefano@gmail.com

**Keywords:** vulvovaginal candidiasis, treatment, therapy, probiotics, vaginal flora, clotrimazole

## Abstract

**Background:** An estimated 75% of women will have one episode of vulvovaginal candidiasis (VCC) during their lifetime, and 40–50% of these will experience further episodes. The high incidence of vulvovaginal candidiasis, combined with the problems of azole resistance and toxicity, highlights the necessity for new strategies for the treatment of this condition. In this context, natural compounds represent promising alternatives. **Methods:** We enrolled, between January 2020 and April 2021, forty women affected by uncomplicated vulvovaginal candidiasis. Women were divided into two groups. In the first group, we treated 20 women with clotrimazole daily administration for six days. In the second group, 20 women were treated with clotrimazole associated with Unilen^®^ Microbio+, a new product containing *Saccharomyces cerevisiae*, melatonin, and GLA-14. Women underwent a check at days 15, 30, and 90. A clinical and cultural examination were performed to establish the effect of the treatments on vaginal flora. **Results:** In the group treated with Unilen^®^ Microbio+, clinical and microbiological cure at 15 and 30 days was observed in 18 women (90%), compared with 16 women (80%) in the group treated only with clotrimazole. The efficacy of the association between clotrimazole and Unilen^®^ Microbio+ in these uncomplicated forms was therefore not inferior to the azole treatment alone. Only four women (20%) in the Unilen^®^ Microbio+ group presented symptomatic recurrences within the 3 months, compared with eight women (40%) in the clotrimazole-only group. Microscopic wet mount analysis at 1 and 3 months demonstrated a significant increase in lactobacillus count and a reduction in the polymorphonucleate cells in the Unilen^®^ Microbio+ group. **Conclusions:** Unilen^®^ Microbio+ supplementation was demonstrated to cure uncomplicated vulvovaginal candidiasis with clotrimazole, reducing recurrence and improving vaginal flora better than clotrimazole treatment alone.

## 1. Introduction

Fungal infection of vulvovaginal environment is the second most common cause of inflammation after bacterial vaginosis. The most common pathogen is *Candida albicans* (*C. albicans*), which is isolated in 85–90% of all cases. An estimated 75% of women will have at least one episode of vulvovaginal candidiasis (VCC) during their lifetime, and 40–50% of these will experience further episodes. Typical symptoms of VVC include pruritus, dyspareunia, internal and external dysuria, abnormal vaginal discharge, and erythematous vulva, but none of this is specific [1].

The high incidence of vulvovaginal candidiasis, combined with the growing problems of azole resistance and toxicity of antifungal drugs, highlights the necessity for the development of new effective strategies for the treatment of this condition. In this context, natural compounds represent promising alternatives [2].

According to the International Scientific Association for Probiotics and Prebiotics (ISAPP), probiotics are defined as “live microorganisms that, when administered in adequate amounts, confer a health benefit to the host”. These organisms provide competition for pathogenic microorganisms and prevent their colonization, thus contributing to host defense mechanisms [3].

The increased vaginal microbiota (VMB) knowledge has led to a renewed interest in using exogenous lactobacilli (probiotics or live biotherapeutic products) to optimize the VMB as part of curative or preventive vaginal dysbiosis interventions. In vitro studies of probiotics have demonstrated that they reduce the virulence of Candida spp. by inhibiting biofilm formation and may provide additional benefit to antifungals. *Lactobacillus rhamnosus* (*L. rhamnosus*), *Lactobacillus casei*, and *Lactobacillus acidophilus* (*L. acidophilus*) significantly reduced levels of *C. albicans* biofilms at the initial colonization phase and the later maturation phase of biofilm development [4].

The leading treatment of vulvovaginal candidiasis (VVC) at the moment is represented by oral or topic azoles. However, often, this type of treatment results in suboptimal cure fractions and high recurrence rates. In addition, prolonged use increases the probability of side effects and drug resistance, complicating the regeneration of lactobacilli. These may not only have a financial impact but can also cause both psychological and physical discomfort, thus affecting the patient’s quality of life and sometimes exposing the woman to more serious complications [5].

Possible mechanisms by which probiotic strains might exert positive effects include niche occupation so that other bacteria cannot expand, and biofilms cannot be established, increased local production or release of lactic acid and other antimicrobial compounds, modulation of local cervicovaginal mucosal immune responses, and/or inhibition of *C. albicans* hyphae formation restoring the equilibrium of the local ecosystem [6]. For this reason, the administration of exogenous lactobacillus preparations for both topical and oral use was proposed and evaluated.

In relation to vulvovaginal candidiasis, evidence published to date reveals that some specific strains, namely, *L. rhamnosus GR-1*, *Lactobacillus fermentum RC-14*, and *Lactobacillus reuteri *(*L. reuteri*)* RC-14*, are effective against *C. albicans* and are a valid alternative to traditional prophylactic antifungal treatments [7]. Numerous in vitro studies have shown that *Lactobacillus delbrueckii*, *Lactobacillus plantarum*, *Lactobacillus acidophilus,* and *Lactobacillus gasseri* can inhibit the adhesion and/or growth of *C. albicans* through the production of biosurfactants or bacteriocin-like substances [8,9].

Analyzing the available studies, it can be seen that the benefits of probiotics are strictly strain-specific and correlated with the type of infection being treated, as well as patient variables (age, hormone status, genetic predisposition, sexual habits, intimate hygiene), the treatment duration, and the type of product used (dose, formulation, route of administration). One line of investigation is what role lactobacilli might have in the restoration of the lactobacillus vaginal flora after conventional antifungal treatments, and if such a therapy might help prevent dysbiosis or recurrent infections. However, little data is available on their effectiveness in formal randomized clinical trials.

In this manuscript, we present our study conducted on 40 women divided into two arms. In the first group, we treated 20 women with a daily administration of topical antifungal treatment with clotrimazole for six consecutive days. In the second group, 20 women were treated with clotrimazole associated with an oral preparation designed to combat uncomplicated forms of vulvovaginal candidiasis by acting as a sort of broad-spectrum supplement, as well as by modulating the inflammatory response, that contained the following active ingredients: *Saccharomyces cerevisiae *(*S. cerevisiae*) live strains, melatonin, and *Lactobacillus acidophilus GLA-14*. The name of this oral preparation is Unilen^®^ Microbio+ (by Uniderm Farmaceutici S.r.l. Viale Enrico Ortolani, 211, 00125 Roma, Italy) and it attempts to employ various routes of attack against the “invasive yeast”.

## 2. Materials and Methods

We enrolled, between January 2020 and April 2021, forty women affected by uncomplicated vulvovaginal candidiasis.

Our inclusion criteria were (1) microscopic diagnosis of vulvovaginal candidiasis through wet mount or vaginal culture, (2) age between 18 and 55 years, but not menopausal women (defined as 12 or more months since last menstrual cycle at appropriate age). Our exclusion criteria were (1) complicated vulvovaginal candidiasis (recurrent and/or occurring in immunosuppressed subject, or chronic candidiasis) (NB: recurrent infection is defined as at least four culturally confirmed episodes in 12 months, infections in immunosuppressed subjects and chronic fungal infections are defined as complicated vulvovaginal candidiasis), (2) systemic antibiotic or antifungal treatment, whether ongoing or in the 4 weeks prior to entering the study, (3) pregnant or breastfeeding women, (4) autoimmune diseases, thyroid diseases, or history of atopy, (5) diabetes mellitus, (6) hypersensitivity to lactobacilli.

Among the 40 women we selected, 85% (34) were found positive for *C. albicans* infection; in the remaining 15% (6), *C. glabrata* was the pathogen of the VVC.

In the first group, we treated 20 women with a daily administration of topical antifungal treatment with clotrimazole for six consecutive days. In the second group, 20 women were treated with Unilen^®^ Microbio+ supplement to clotrimazole treatment.

The primary endpoints of our study were as follows:

- Evaluation of the efficacy of the supplementation of Unilen^®^ Microbio+^®^ in women with acute VVC, considering:
  1. Clinical cure rate (resolution of symptoms) of VVC at 15, 30 and 90 days.
  2. Microbiological and microscopic cure of VVC (negative vaginal culture or absence of hyphae at wet mount).
  3. Recurrence rate (defined as the presence of symptoms or positive fungal culture or presence of hyphae at wet mount) in 3-month follow-up period.
  4. Re-equilibrium of the vaginal microflora (through assessment of clinical signs and lactobacillus grade) in women with uncomplicated vulvovaginal candidiasis.

Secondary outcomes:

- Safety and tolerability of Unilen^®^ Microbio+ in adult women.

Women started therapy at the diagnosis of vulvovaginal candidiasis. Then, they underwent a check at days 15, 30, and 90. At each visit, the patient was asked if she had taken the treatment regularly and if she had experienced any clinical symptoms or adverse events. A clinical examination, microscopic wet mount examination, and a cultural examination were performed to establish the effect of the treatment and the lactobacillus grade.

In the group of women treated with clotrimazole, patients underwent a therapy with topic clotrimazole at 2% for six days. The vaginal application of the cream was performed at night before sleeping.

In the group of Unilen^®^ Microbio+, patients underwent a supplement therapy with a night and day formula involving the administration of two different daily formulas for 15 days:

- 1 tablet containing Saccharomyces cerevisiae in the morning.
- 1 tablet containing melatonin and GLA-14 in the evening.

This differentiation aimed to optimize the multiple functions of the various active ingredients in harmony with the circadian rhythm, which can be disturbed by worsening of the infection at night.

The study was conducted according to the guidelines of the Declaration of Helsinki, and approved by the Institutional Review Board of Institute for Maternal and Child Health IRCCS “Burlo Garofolo”, Trieste (IRB:RC 08/2020 15 April 2020).

## 3. Results

In the group treated with Unilen^®^ Microbio+ supplement, clinical and microbiological cure at 15 and 30 days was observed in 18 women (90%), compared with 16 women (80%) in the group treated with topical clotrimazole only. In these uncomplicated forms, the association between the azole and Unilen^®^ Microbio+ was more effective than the azole treatment alone.

Only four women (20%) in the Unilen^®^ Microbio+ group presented symptomatic recurrences within the 3 months of follow-up, compared with eight women (40%) in the clotrimazole-only group. No recurrence was found in the six women with *C. glabrata* VVC.

**Figure 1:**
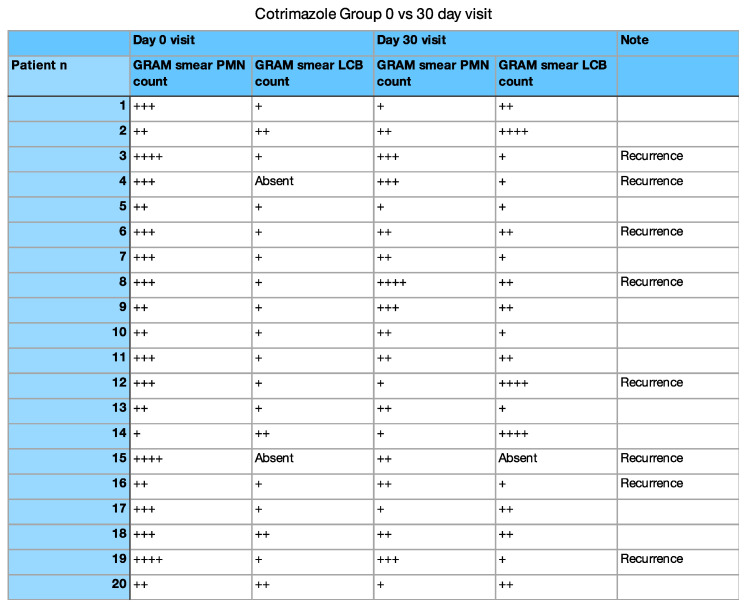
Cotrimazole group GRAM smear results. (+ stands for average number of cells seen per oil immersion field).

**Figure 2:**
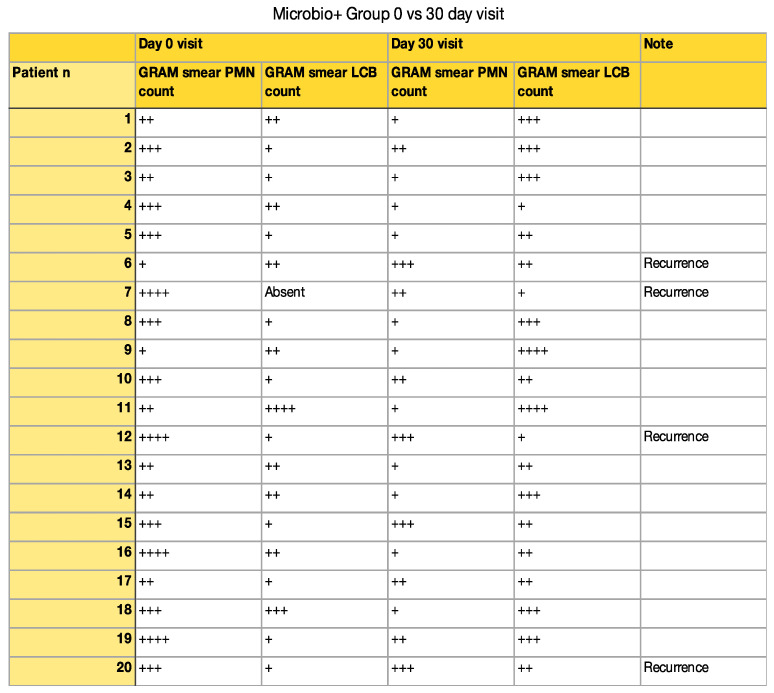
Microbio+ group GRAM smear results. (+ stands for average number of cells seen per oil immersion field).

Microscopic wet mount analysis at 1 and 3 months demonstrated a significant increase in lactobacillus count and a reduction in the polymorphonucleate cells in the Unilen^®^ Microbio+ group. More specifically, evaluating the GRAM stained vaginal smears obtained at the 30 day visit, it was observed that the lactobacillus count before treatment was 1+ (where the + stands for average number of cells seen per oil immersion field [10]) or absent, and after the treatment was mostly 2+/3+. On the other hand, polymorphonucleate cells count was 3+ before treatment and was found mainly at 1+ after treatment with Unilen^®^ Microbio+ (Figure 1 and Figure 2).

All women tolerated the therapy well without any adverse effects for the duration of treatment.

No significative differences in tolerance and recurrence among women of different age were observed in the Microbio group and in the Clotrimazole group

This result supports the concept that the use of Unilen^®^ Microbio+ supplement determines not only cure of uncomplicated fungal infections equivalent to the topical azole alone, but also the early (1 month) and long-lasting (3 months) re-equilibration of the vaginal microflora, reducing the fungal infection recurrence rate. This effect could be ascribed both to the ability of Unilen^®^ Microbio+ to modulate the inflammatory response (reduction in polymorphonucleates) and to the significant increase in the vaginal lactobacillus flora (booster effect).

## 4. Discussion

As vulvovaginal candidiasis is one of the most frequent vulvovaginal infections, it becomes clear that a suitable treatment must be found. The need for alternative treatment approaches arises from the frequent finding, in clinical practice, of recurrent infections. The failure of the antimicrobial treatments commonly used to treat these infectious diseases is related to two main factors. First, the improper use of over-the-counter medicines as self-medication, as increasingly advertised on blogs and forums targeted at women, has led to the development of antimicrobial resistance. Second, the negative impact of antimicrobials themselves on the vaginal microflora complicates the regeneration of lactobacilli—the vaginal ecosystem’s first line of defense in women of reproductive age. It has been shown that the typical vaginal microflora of the reproductive age is dominated at 95% by lactobacilli. These microorganisms help boost the local immune defenses through the creation of a biofilm which prevents the adhesion of pathogens, competition for metabolites, and the production of various chemicals, such as hydrogen peroxide, bacteriocin, and lactic acid. Recent years have seen a rise in the number of studies investigating the efficacy of probiotics in the treatment and/or prevention of infections of the female lower urogenital tract, especially vulvovaginal candidiasis and bacterial vaginosis (BV), the most common infections in women of reproductive age. Variable results have been obtained in the application of lactobacilli to VVC treatment, mostly due to the many variables that have to be taken into account. This difficulty is well shown by the review of the literature on this subject between 1975 and 2006 made by Falagas et al., where no univocal results were drawn due to the many variables between the studies [11]. On the other hand, when a specific strain of lactobacilli is studied, the results demonstrate an overall benefit in term of resolution of acute symptoms and reduction of relapses, as shown by Martinez et al., with the supplementation of lactobacilli capsules (containing *L. rhamnosus GR-1* and *L. reuteri RC-14*) for 4 weeks after an initial single dose of fluconazole treatment that led to an improved clinical and cultural result with less symptoms for the patient, and lower presence of yeast detected by culture, and by De Seta et al. with *L. plantarum P17630* with similar results in the treatment of acute VVC [12,13].

More recent metanalysis conducted by Jeng et al. in 2020 demonstrated that probiotic treatments are useful for managing common vaginal infections, particularly BV and VVC. The results of the meta-analysis indicated that probiotics as a supplement of antibiotic/anti-fungal treatments reduced the recurrence rate and increased the cure/remission rate in nonpregnant adult females at 1 month after treatment. The outcome supports the effectiveness of Lactobacilli in decreasing the recurrence rate and improving the cure rate [14].

Some studies have shown a correlation between *S. cerevisiae* and recurrent VVC [15]. It was also shown that prolonged use of probiotic medications containing *S. cerevisiae* could lead to an overgrowth of the microbe in the gastrointestinal tract [16]. Therefore, it could be hypothesized that using this type of probiotic could aggravate the vulvovaginal candidiasis recurrence rate, causing worse symptoms in the patients. No overcolonization by *S. cerevisiae* in the vaginal flora was observed during our study.

As for safety, the majority of evidence has shown that probiotics are safe to recommend to patients. Some complications indicated are sepsis, fungemia, and GI ischemia. Still, the risk of adverse effects usually interests critically ill patients in intensive care units, critically sick infants, postoperative and hospitalized patients, and patients with immunocompromised complexity [17].

The strength of our study is based on the presence of two homogeneous groups of patients from the point of view of the vaginal flora before therapy, and on the use of a new product for the treatment of uncomplicated vulvovaginal candidiasis.

The limitations are based on the presence of a small sample of patients in the two groups. The current work has a power <0.5 with alpha set at 0.1; future efforts should target a power of 0.9, alpha = 0.05, thus needing 105 subjects in each group. Another limitation is represented by the observation of recurrence only during the treatment period and the fact that no long-term data about the lactobacilli and polymorphonucleate vaginal population count were obtained. This should be considered as a preliminary study. It is always difficult to initially test a new product on a large sample of the population. However, we intend to expand the sample and long-term evaluation on the vaginal microbiota in the future.

## 5. Conclusions

In the present study, we compared the effectiveness of clotrimazole alone versus the use of Unilen^®^ Microbio+ supplement therapy. Both groups had a high percentage of clinical and cultural response to the treatment; however, we observed that in the clotrimazole-only group, the recurrence rate was twice higher than the group that underwent Unilen^®^ Microbio+ therapy. Moreover, when analyzing the vaginal flora of the patients treated with Unilen^®^ Microbio+, we observed a significant increase in lactobacillus count and a reduction in the polymorphonucleate cells (Figure 2). Therefore, it is safe to believe that Unilen^®^ Microbio+ works through the re-equilibration of the vaginal microflora, eventually reducing the fungal infection recurrence rate.

## Data Availability

The original contributions presented in the study are included in the article; further inquiries can be directed to the corresponding author.
